# Impact of perioperative low-molecular-weight heparin therapy on clinical events of elderly patients with prior coronary stents implanted > 12 months undergoing non-cardiac surgery: a randomized, placebo-controlled trial

**DOI:** 10.1186/s12916-024-03391-2

**Published:** 2024-04-23

**Authors:** Bin Wang, Yanhui Su, Cong Ma, Lining Xu, Qunxia Mao, Wenjia Cheng, Qingming Lu, Ying Zhang, Rong Wang, Yan Lu, Jing He, Shihao Chen, Lei Chen, Tianzhi Li, Linggen Gao

**Affiliations:** 1 Department of Comprehensive Surgery, The Second Medical Center & National Clinical Research Center for Geriatric Diseases, Chinese PLA General Hospital, Beijing, 100853 China; 2 Health Management Institute, The Second Medical Center & National Clinical Research Center for Geriatric Diseases, Chinese PLA General Hospital, Beijing, China; 3 National Research Institute for Family Planning, Beijing, China; 4 Department of Thoracic Surgery of The First Medical Center, General Hospital of Chinese People’s Liberation Army, Beijing, 100853 China

**Keywords:** Perioperative, Percutaneous coronary intervention, Bridging therapy, Low-molecular-weight heparin, Clinical event

## Abstract

**Background:**

Little is known about the safety and efficacy of discontinuing antiplatelet therapy via LMWH bridging therapy in elderly patients with coronary stents implanted for > 12 months undergoing non-cardiac surgery. This randomized trial was designed to compare the clinical benefits and risks of antiplatelet drug discontinuation via LMWH bridging therapy.

**Methods:**

Patients were randomized 1:1 to receive subcutaneous injections of either dalteparin sodium or placebo. The primary efficacy endpoint was cardiac or cerebrovascular events. The primary safety endpoint was major bleeding.

**Results:**

Among 2476 randomized patients, the variables (sex, age, body mass index, comorbidities, medications, and procedural characteristics) and percutaneous coronary intervention information were not significantly different between the bridging and non-bridging groups. During the follow-up period, the rate of the combined endpoint in the bridging group was significantly lower than in the non-bridging group (5.79% vs. 8.42%, *p* = 0.012). The incidence of myocardial injury in the bridging group was significantly lower than in the non-bridging group (3.14% vs. 5.19%, *p* = 0.011). Deep vein thrombosis occurred more frequently in the non-bridging group (1.21% vs. 0.4%, *p* = 0.024), and there was a trend toward a higher rate of pulmonary embolism (0.32% vs. 0.08%, *p* = 0.177). There was no significant difference between the groups in the rates of acute myocardial infarction (0.81% vs. 1.38%), cardiac death (0.24% vs. 0.41%), stroke (0.16% vs. 0.24%), or major bleeding (1.22% vs. 1.45%). Multivariable analysis showed that LMWH bridging, creatinine clearance < 30 mL/min, preoperative hemoglobin < 10 g/dL, and diabetes mellitus were independent predictors of ischemic events. LMWH bridging and a preoperative platelet count of < 70 × 10^9^/L were independent predictors of minor bleeding events.

**Conclusions:**

This study showed the safety and efficacy of perioperative LMWH bridging therapy in elderly patients with coronary stents implanted > 12 months undergoing non-cardiac surgery. An alternative approach might be the use of bridging therapy with half-dose LMWH.

**Trial registration:**

ISRCTN65203415.

**Supplementary Information:**

The online version contains supplementary material available at 10.1186/s12916-024-03391-2.

## Background

An increasing number of patients are prescribed single antiplatelet therapy for the prevention of myocardial infarction (MI) and coronary stent thrombosis more than 12 months after the placement of bare metal stents (BMSs) and drug-eluting stents (DESs) [1, 2]. Patients with coronary stents who are taking antiplatelet drugs and who require non-cardiac surgery or invasive procedures are commonly encountered, and their perioperative management is an important consideration.

In patients who undergo percutaneous coronary intervention (PCI) with stenting, there is a delicate balance between the risk of cardiovascular or thrombotic events and the potential risk of bleeding complications. The impact of discontinuing antiplatelet agents in patients with implanted stents undergoing non-cardiac surgery is debated, with previous studies showing conflicting results [3, 4]. Discontinuation of antiplatelet therapy has the potential to increase the risk of major perioperative adverse cardiovascular events, with stent thrombosis being the most feared because of its high associated morbidity and mortality [3, 5]. The continuation of antiplatelet therapy is recommended for patients who are at a moderate-to-high risk of cardiovascular events, while discontinuation is recommended in low-risk patients [6]. However, in the POISE-2 trial, perioperative use of aspirin had no significant effect on the combined risk of death or non-fatal MI in patients undergoing non-cardiac surgery, but it did increase the risk of bleeding [7].

Bridging therapy with low-molecular-weight heparin (LMWH) is usually recommended in patients who require interruption of anticoagulation before surgery [8–10]. Although platelets are the primary players in stent thrombosis, coagulation also plays a role [11]. To avoid thrombotic events during surgery while minimizing the perioperative bleeding risk, clinicians often administer LMWH in clinical practice to “bridge” patients undergoing non-cardiac surgery until the previous antiplatelet treatment can be resumed [4, 12]. However, in patients with stents who require bridging via non-cardiac surgery, the European Society of Cardiology guidelines discourage the use of LMWH because this approach might be associated with a greater bleeding risk [13]; however, this approach still needs to be validated in a randomized clinical study.

With aging, the onset of comorbidities and impairments in cardiac, renal, and hepatic function not only increase the incidence of perioperative ischemic cardiovascular or thrombotic events, but they also increase the risk of major bleeding. The optimal management of these complex patients has [14] not been determined, and data from randomized controlled trials are lacking. Randomized controlled trials comparing the use of bridging therapy with no bridging therapy in patients with atrial fibrillation have shown a higher bleeding risk without a change in the incidence of thromboembolic events [15, 16]. Increasing concern has been raised that bridging therapy increases the risk of bleeding in patients without reducing the risk of thromboembolism. However, little is known about the safety and efficacy of discontinuing antiplatelet therapy via LMWH bridging therapy in older patients with coronary stents implanted for > 12 months undergoing non-cardiac surgery. The present randomized controlled trial aimed to shed some light on this debated topic.

## Methods

This study was reported using the Consolidated Standards of Reporting Trials (CONSORT) guidelines [17] (Additional File 1: CONSORT Checklist).

### Study design and oversight

The trial (ISRCTN65203415) was a randomized placebo-controlled trial. The study was approved by the ethics committee of Chinese PLA General Hospital. The clinical coordinating group was responsible for study coordination, patient randomization, and therapy assignment. The data coordinating group was responsible for maintenance of the study database, data validation, and data analysis.

### Patients

Patients who met the following inclusion criteria were included in the trial: (1) ≥ 75 years of age, (2) underwent PCI with stents > 12 months before non-cardiac surgery, (3) treated with antiplatelet therapy for ≥ 1 year, and (4) were undergoing elective surgery or other elective invasive procedures that required interruption of antiplatelet therapy.

Patients’ characteristics included general demographics, clinical covariates, laboratory values, comorbidities, type of surgery, perioperative medication, and PCI information. The surgical procedures were categorized according to surgical risk based on the definition of the Revised Cardiac Risk Index (RCRI) [18].

**Fig. 1:**
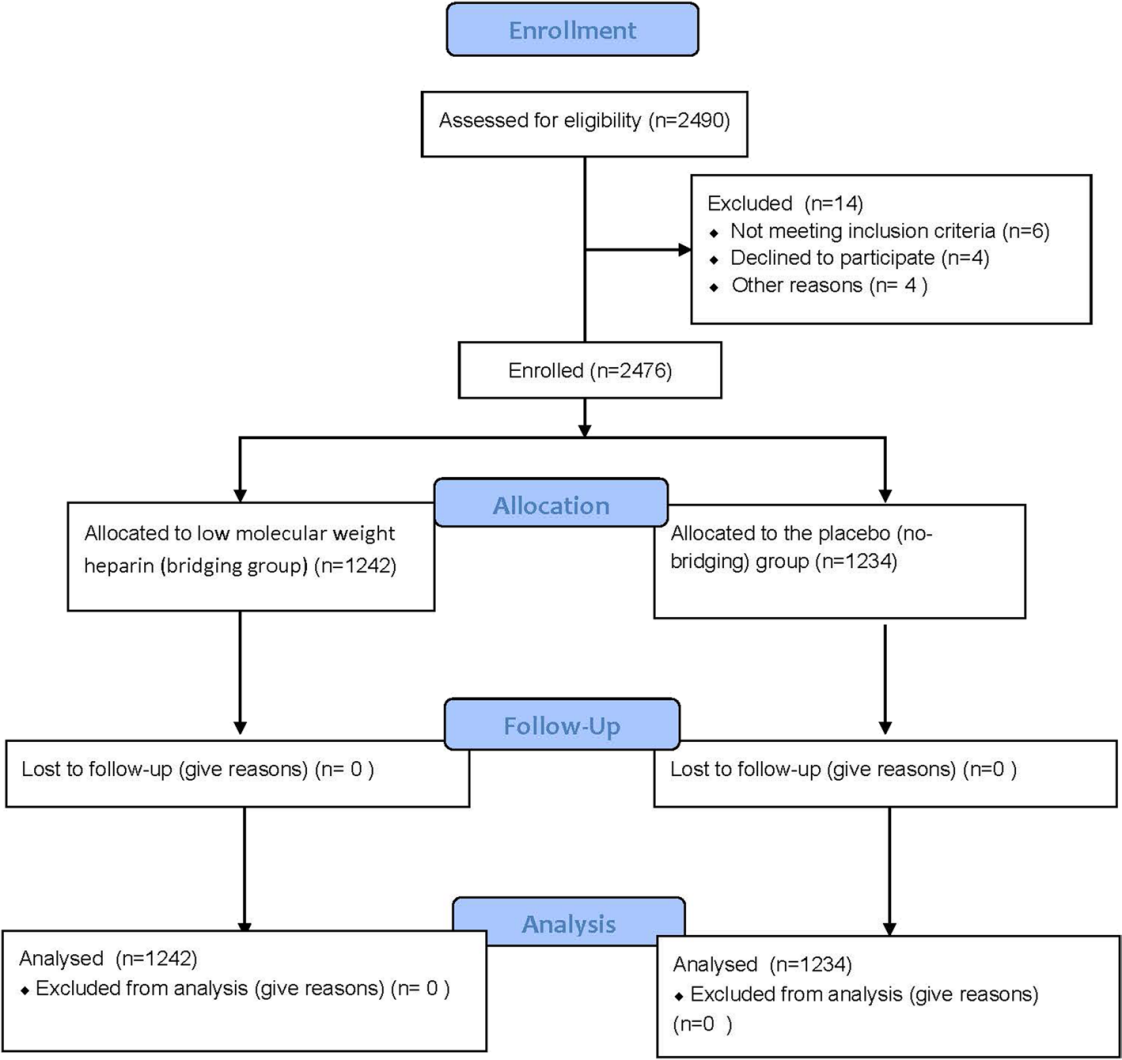
An overview of selection of study participants

Patients were excluded if they met one or more of the following: (1) < 75 years of age; (2) had taken antiplatelet therapy for < 12 months; (3) were scheduled for surgery with local anesthesia; (4) were planned for cardiac surgery; (5) had major cardiac ischemic events and/or bleeding within the previous 6 weeks; (6) had a mechanical heart valve, some of whom were receiving both oral anticoagulant therapy and antiplatelet therapy; (7) had a platelet count of < 100 × 10^3^/mm^3^. The patients were recruited from Chinese PLA General Hospital, and all patients provided written informed consent. Figure 1 shows the patient selection process.

### Procedures

Randomization was performed using a computer-generated random number list in a 1:1 ratio in block sizes of six patients. The patients were randomly assigned to receive LMWH bridging therapy with dalteparin sodium (2500 IU administered subcutaneously twice daily) or no bridging therapy (a matching subcutaneous placebo). Dalteparin sodium and placebo were prepared by the pharmacist. A randomized sequence list was also constructed in which the subject number was linked to the study medication number. Gao G.L., Mao Q.X., Li T.Z., Su Y.H., and Ma C. generated the random allocation sequence; Xu L.N., Cheng W.J., Wang R., Lu Q.M., Zhang Y., Wang R., and Lu Y. enrolled the patients; and Wang B., He J., Chen S.H., and Chen L. assigned the patients to the interventions.

**Fig. 2:**
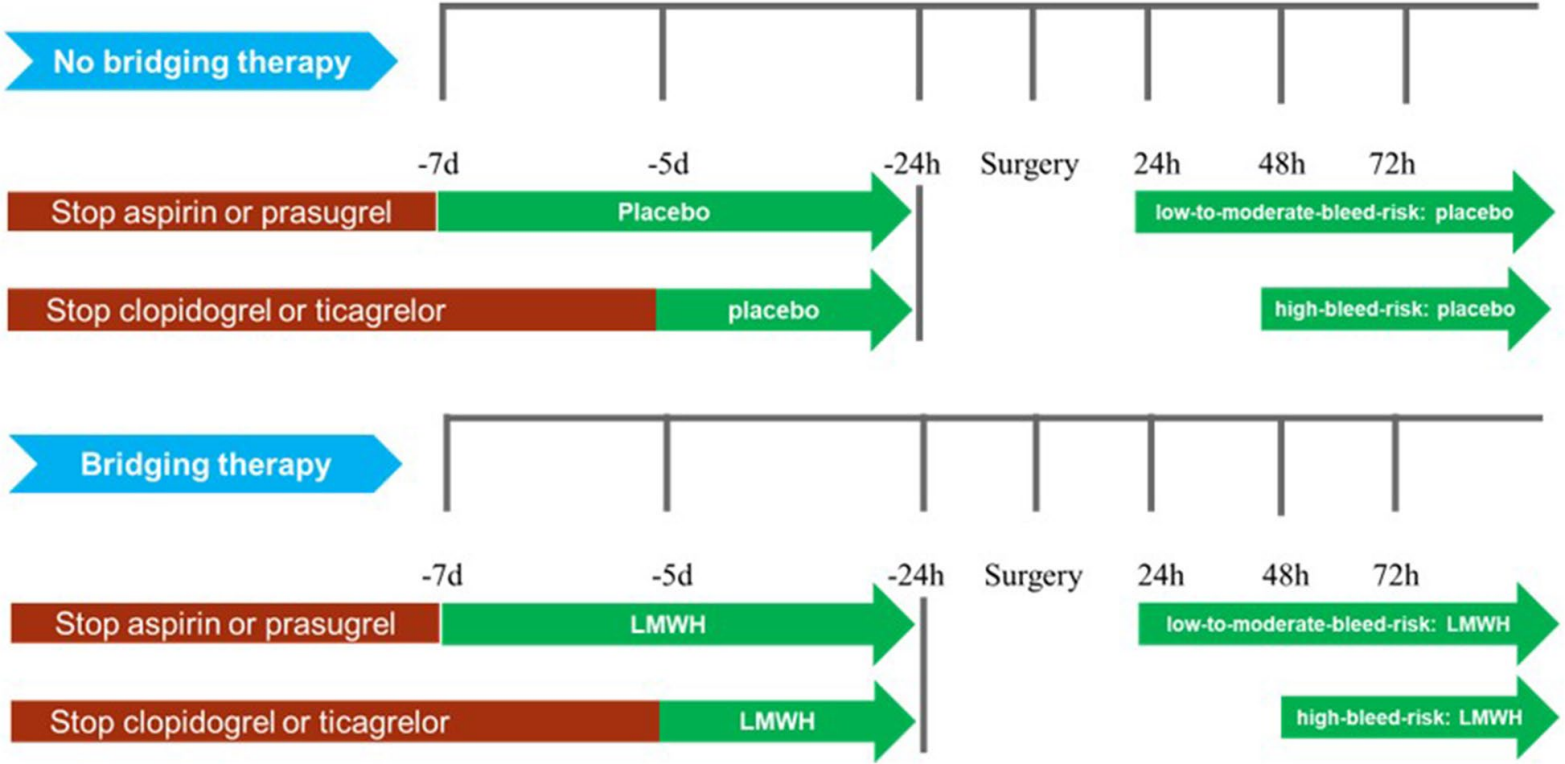
Study design

In patients requiring antiplatelet interruption, aspirin and prasugrel were interrupted for 7 days, and clopidogrel and ticagrelor were interrupted for 5 days before the elective surgery/procedure. The last preoperative dose of LMWH was administered 24 h before surgery [19]. LMWH or placebo was resumed 24 h after a surgery/procedure with a low-to-moderate bleeding risk and 48–72 h after a surgery/procedure with a high bleeding risk [6] (Fig. 2). The procedural bleeding risk was assessed based on International Society on Thrombosis and Haemostasis (ISTH) guidance statements [14]. The patients were divided into “high,” “low-to-moderate,” and “minimal” bleeding risk categories based on the expected 30-day postoperative risk of major bleeding (high bleeding risk: ≥ 2%, low-to-moderate bleeding risk: 0–2%, and minimal bleeding risk: 0%) [20].

### Data collection

Clinical and surgical variables and laboratory data were collected using standardized reporting forms and assessed for quality. Clinical and surgical variables included baseline characteristics and PCI information of the study population. The following laboratory measurements were collected: hemoglobin, activated partial thromboplastin time (APTT), creatine kinase (CK), CK-MB, myoglobin, lactate dehydrogenase, creatinine, albumin, sodium, N-terminal pro-brain natriuretic peptide (NT-proBNP), high-sensitivity cardiac troponin T (hs-cTnT). Biochemical measurements were performed using standard laboratory techniques. CK-MB level was measured by the Abbott AXSYM automatic immune analyzer (Abbott Laboratories, Chicago, IL). Hs-cTNT, creatinine, and NT-proBNP were measured by the Cobas 8000 instrument. NT-proBNP and hs-cTNT were analyzed by the electrochemiluminescence immunoassays “ECLIA.”

Clinical follow-up data were prospectively collected through scheduled clinic evaluations. The patients were followed up by telephone each week, with the final telephone follow-up conducted 30 days after the procedure.

### Study outcomes

All study outcomes were assessed 30 days after the procedure. The primary efficacy endpoint was the composite of ischemic cardiac or cerebrovascular events, defined as perioperative myocardial injury, perioperative MI, cardiac death, and non-fatal ischemic stroke. Perioperative myocardial injury was defined as a postoperative hs-cTnT concentration of 20 to < 65 ng/L, with an absolute change of at least 5 ng/L or an hs-cTnT concentration of ≥ 65 ng/L within 3 days after non-cardiac surgery, without any of the clinical, electrocardiogram (ECG), or imaging criteria [21–24]. Perioperative acute MI was diagnosed if the hs-cTnT criteria for perioperative myocardial injury were met and accompanied by one or more of the following: ischemic symptoms (e.g., chest pain), ischemic ECG changes, new regional wall motion abnormalities, or coronary thrombus [21–24]. Non-fatal stroke was defined as any ischemic cerebrovascular disease.

The primary safety outcome was major bleeding defined by one or more of the events defined by the ISTH [15]. The secondary efficacy endpoints were pulmonary embolism, deep vein thrombosis, and death. The secondary safety endpoint was minor bleeding. All study endpoints were independently and blindly adjudicated.

### Statistical analysis

The sample size was determined to achieve a precise estimate of the efficacy and safety of the protocol. According to a previous systematic review, the estimated risk of periprocedural thromboembolic events is 0.89% (≈0.9%) and 0.46% (≈0.5%) for the bridged and non-bridged groups, respectively [16]. A sample size of 2384 patients was considered appropriate for the present study because it would yield a 1.0% margin of error at the 95% confidence level (two-sided significance level = 0.05). This sample size would give the study 80% power to detect the expected difference in the rate of periprocedural thromboembolic events. With a 5% allowance for patient withdrawal from the study, the required sample size was 2500 patients. The sample size was calculated online (https://powerandsamplesize.com).

Patients’ data are summarized using descriptive statistics. The normality of the continuous numeric variables is tested before analysis. The data in normal distribution are reported as the mean ± standard deviation and are compared between the bridged and non-bridged groups using Student’s *t*-test. And the data in non-normal distribution are expressed in quartiles: P50 (P25; P75). The Mann-Whitney *U* test is used to compare the differences between the two groups. Categorical variables are presented as count (percentage) and were compared between the two groups using the chi-square test or Fisher’s exact test.

The primary efficacy endpoint was the composite of ischemic cardiac or cerebrovascular events at 30 days after the procedure. The primary safety endpoint was major bleeding at 30 days after the procedure. To identify the predictors of clinical outcomes, a multivariable analysis was performed via stepwise logistic regression. Candidate variables or covariates included in the logistic regression model: age (continuous variable), sex (male/female), LMWH bridging, American Society of Anesthesiologists (ASA), body mass index (BMI), medications, comorbidities, type of surgery, preoperative hemoglobin, preoperative platelet, perioperative mean arterial pressure (MAP), creatinine clearance. The results of logistic regression are presented as relative risk (RR) and 95% confidence intervals (CIs). We considered *p* < 0.05 as statistically significant, and the SPSS 26.0 software was used for all statistical analyses.

## Results

### Patients’ characteristics

We recruited 2490 patients between January 1, 2023, and November 1, 2023. Of the 2490 patients enrolled in the trial, 14 patients did not participate. The patients’ baseline characteristics are presented in Table 1. A total of 1242 patients were assigned to receive LMWH bridging therapy (bridging group), and 1234 patients were assigned to the placebo (non-bridging) group. The demographic and baseline variables (sex, age, body mass index, comorbidities, RCRI score, medications, procedural characteristics, and preoperative hs-cTnT concentration) were not significantly different between the two groups. The characteristics were well balanced between the two groups.

**Table 1:** Baseline characteristics

	All patients (*N* = 2476)	Bridging therapy (*n* = 1242)	No bridging therapy (*n* = 1234)	*P* value
General demographics				
Age	82.58 ± 6.75	82.33 ± 6.80	82.84 ± 6.70	0.060
Sex, male, *n* (%)	2036 (82.2)	1006 (81.0)	1030 (83.4)	0.115
ASA, *n* (%)				
I	100 (4.04)	52 (4.19)	48 (3.89)	
II	1710 (69.06)	850 (68.44)	860 (69.69)	
III	522 (21.08)	270 (21.74)	252 (20.42)	
IV	144 (5.82)	70 (5.64)	74 (6.00)	0.453
≥ 4MET, *n* (%)	1580 (63.81)	808 (65.06)	772 (62.56)	0.196
Clinical covariates				
Body mass index, kg/m^2^	24.3 ± 2.5	23.9 ± 2.9	24.8 ± 2.1	0.746
Antiplatelet drugs				
Aspirin use, *n* (%)	1975 (79.77)	980 (78.90)	995 (80.63)	0.285
Clopidogrel use, *n* (%)	386 (15.59)	194 (15.62)	192 (15.56)	0.967
Prasugrel, *n* (%)	80 (3.23)	45 (3.62)	35 (2.84)	0.268
Ticagrelor, *n* (%)	35 (1.41)	23 (1.85)	12 (0.97)	0.064
ACE inhibitor or ARB use, *n* (%)	882 (35.62)	447 (35.99)	435 (35.25)	0.701
Statin use, *n* (%)	1922 (77.58)	958 (74.88)	964 (80.31)	0.556
Comorbidities				
Prior myocardial infarction, *n* (%)	584 (23.6)	278 (22.38)	306 (24.79)	0.157
Chronic heart failure, *n* (%)	566 (28.60)	269 (21.65)	297 (24.06)	0.153
Chronic kidney disease, *n* (%)	616 (24.87)	291 (23.42)	325 (26.33)	0.094
Atrial fibrillation, *n* (%)	358 (14.50)	195 (15.70)	163 (13.21)	0.078
Peripheral artery disease, *n* (%)	992 (40.10)	482 (38.81)	510 (41.32)	0.201
Hypertension, *n* (%)	1328 (53.63)	654 (52.67)	674 (54.61)	0.328
Prior stroke/TIA, *n* (%)	737 (29.76)	378 (30.43)	359 (29.09)	0.465
Diabetes mellitus, *n* (%)	1306 (52.70)	659 (53.05)	647 (52.43)	0.754
COPD, *n* (%)	870 (35.10)	421 (33.90)	449 (36.39)	0.195
Asthma, *n* (%)	289 (11.70)	150 (12.08)	139 (11.26)	0.529
ARISCACT score	27 ± 7	26 ± 8	28 ± 6	0.536
Active tumor disease, *n* (%)	814 (32.87)	395 (31.80)	419 (33.95)	0.255
RCRI score, *n* (%)				
I	214 (8.64)	113 (9.10)	101 (8.18)	0.419
II	1267 (51.20)	647 (52.09)	620 (50.24)	0.357
III	324 (13.1)	175 (14.09)	149 (12.07)	0.137
IV	671 (27.10)	307 (24.72)	364 (29.50)	0.007
Type of surgery, *n* (%)				
Gastrointestinal Surgery	636 (25.69)	311 (25.04)	325 (26.33)	0.460
Thoracic	337 (13.61)	159 (12.80)	178 (14.42)	0.239
Hepatobiliary surgery	318 (12.84)	166 (13.67)	152 (12.32)	0.436
Orthopedic	225 (9.09)	119 (9.58)	106 (8.59)	0.391
Cerebral surgery	192 (7.75)	97 (7.81)	95 (7.70)	0.917
Urology	709 (28.63)	359 (28.90)	350 (28.36)	0.765
Other	59 (2.38)	31 (2.50)	28 (2.27)	0.711
High-bleed-risk surgery , *n* (%)	2450 (98.95)	1230 (99.03)	1220 (98.87)	0.681
Low-to-moderate-bleed-risk surgery (30-day risk of major bleed 0–2%), *n* (%)	18 (0.73)	7 (0.56)	11 (0.89)	
Minimal-bleed-risk surgery (30-day risk of major bleed approximately 0%), *n* (%)	8 (0.32)	5 (0.41)	3 (0.24)	
Laboratory values				
Hemoglobin, g/dl	121.96 ± 18.39	122.88 ± 17.63	121.04 ± 19.17	0.413
Preoperative platelet (×10^9^/L)	189.59 ± 58.88	191.31 ± 58.82	187.85 ± 58.91	0.547
Perioperative MAP, mmHg	67.53 ± 7.24	66.75 ± 6.29	68.33 ± 7.56	0.511
APTT, s	35.53 ± 6.50	35.42 ± 6.69	35.64 ± 6.37	0.619
D-dimer, mg/ml	1.02 (0.47,2.03)	1.06 (0.50,2.18)	1.00 (0.44.1.95)	0.130
C-reactive protein, mg/l	1.46 (0.33,4.73)	1.44 (0.33,4.71)	1.51 (0.35,4.92)	0.864
Creatine kinase, U/l	62.00 (31.50,109.2)	64.00 (32.10,113.7)	60.35 (30.30,109.20)	0.150
Bilirubin, μmol/l	18.05 ± 2.85	18.32 ± 3.55	17.78 ± 1.55	0.945
CK-MB, U/l	24.46 ± 5.90	25.40 ± 7.40	23.52 ± 4.31	0.587
Myoglobin, U/l	45.00 (33.00,67.00)	45.50 (32.00,69.25)	43.00 (31.00,63.00)	0.437
Lactate dehydrogenase, U/l	2.37 ± 0.78	2.37 ± 0.82	2.38 ± 0.75	0.910
Creatinine, mg/dl	78.54 ± 31.42	77.80 ± 30.81	79.30 ± 32.05	0.785
Albumin, g/dl	33.34 ± 4.80	32.73 ± 5.19	33.95 ± 4.74	0.708
Sodium, mEq/l	139.66 ± 3.54	139.52 ± 3.43	139.80 ± 3.64	0.920
BNP, ng/ml	335.76 ± 27.41 ±	347.88 ± 19.01	323.56 ± 35.80	0.859
Preoperative hs-cTnT, ng/l	17.00 (10.00,31.00)	19.00 (10.00,30.00)	16.00 (10.00,35.00)	0.164
Maximum Postoperative hs-cTnT, ng/l	20.00 (14.00,38.00)	20.00 (10.00,40.00)	24.00 (14.00.,34.00)	0.000

*ACEI* angiotensin-converting enzyme inhibitor, *ARB* angiotensin receptor blocker, *ASA* American Society of Anesthesiologists, *APTT* activated partial thromboplastin time, *BNP* brain natriuretic peptides, *CK-MB* creatine kinase-MB, *COPD* chronic obstructive pulmonary disease, *MAP* mean artery pressure, *METS* metabolic equivalent of task, *RCRI* Revised Cardiac Risk Index

Of the 2476 patients enrolled in the trial, 1975 (79.77%) were treated with aspirin, and 386 (15.59%) were treated with clopidogrel. Table 2 shows the PCI information of the study population. Compared with the bridging group, the non-bridging group had a significantly higher proportion of patients treated for two-vessel disease (46.76% vs. 41.94%, *p* = 0.016) and a lower proportion of patients treated for single-vessel disease (39.14% vs. 45.00%, *p* = 0.003). There was no significant difference in the type of stent used for PCI between the two groups (*p* = 0.471). The maximum postoperative hs-cTnT concentration in the bridging group was significantly lower than in the non-bridging group (*p* = 0.000).

**Table 2:** PCI information of the study population

	All patients (*N* = 2476)	Bridging therapy (*n* = 1242)	No bridging therapy (*n* = 1234)	*P* value
PCI target vessel				
Left main, *n* (%)	27 (1.1)	12 (0.97)	15 (1.22)	0.550
Left anterior descending artery, *n* (%)	818 (33.04)	421 (33.90)	397 (32.17)	0.361
Right coronary artery, *n* (%)	953 (38.49)	498 (40.10)	455 (36.87)	0.057
Circumflex, *n* (%)	487 (19.67)	233 (18.76)	254 (20.58)	0.254
Other, *n* (%)	512 (20.68)	259 (20.85)	253 (20.50)	0.829
Number of vessels treated per patient				
One vessel, *n* (%)	1042 (42.08)	559 (45.0)	483 (39.14)	0.003
Two vessels, *n* (%)	1098 (44.35)	521 (41.94)	577 (46.76)	0.016
Three, *n* (%)	196 (7.92)	95 (3.62)	91 (3.32)	0.796
Other, *n* (%)	140 (5.65)	67 (5.39)	73 (5.92)	0.575
Number of stents implanted per patient, *n* (%)	2.20 ± 1.32	2.06 ± 1.25	2.35 ± 1.42	0.416
BMS	250 (10.10)	120 (9.66)	130 (10.53)	0.471
DES	2226 (89.90)	1122 (90.34)	1104 (89.47)	
Mean time interval between PCI and surgery ,months	76.22 ± 29.01	80.88 ± 20.45	71.53 ± 35.80	0.348

*PCI* percutaneous coronary intervention, *DES* drug-eluting stent, *BMS* bare metal stent

### Perioperative anticoagulant management

The mean number of drugs administered was 5.69 ± 2.02. There was no significant difference in the mean number of drugs used between the non-bridging group and the bridging group. Patients in the bridging group were administered dalteparin sodium 2500 IU subcutaneously twice daily. Patients who experienced bleeding in the perioperative period required interruption of LMWH therapy. Antiplatelet treatment was restarted 72 h after a surgery/procedure with a low-to-moderate bleeding risk and 7 days after a surgery/procedure with a high bleeding risk at the patient’s usual dose.

### Thirty-day ischemic and bleeding outcomes

All of the enrolled patients completed the study and provided clinical outcome data. The perioperative clinical outcomes of the patients are shown in Table 3. During the follow-up period, the rate of the combined endpoint in the bridging group was significantly lower than in the non-bridging group (5.79% vs. 8.42%, *p* = 0.012). The incidence of myocardial injury in the bridging group was significantly lower than in the non-bridging group (3.14% vs. 5.19%, *p* = 0.011). There was no significant difference between the groups in terms of the rates of acute MI, cardiac death, stroke, and major bleeding. The median time to a major bleeding outcome after the procedure was 5 days (interquartile range 1.0–15.0 days).

**Table 3:** Perioperative clinical outcomes

Variable	Bridging (*N* = 1242)	No bridging (*N* = 1234)	*P* value
**Primary endpoints**			
Myocardial injury, *n* (%)	39 (3.14)	64 (5.19)	0.011
Acute myocardial infarction, *n* (%)	10 (0.81)	17 (1.38)	0.170
Cardiac death, *n* (%)	3 (0.24)	5 (0.41)	0.473
Stroke, *n* (%)	2 (0.16)	3 (0.24)	0.649
Major bleeding, *n* (%)	18 (1.45)	15 (1.22)	0.612
The combined primary endpoint	72 (5.79)	104 (8.42)	0.012
**Secondary endpoints**			
Death, *n* (%)	6 (0.48)	8 (0.65)	0.584
Deep-vein thrombosis, *n* (%)	5 (0.40)	15 (1.21)	0.024
Pulmonary embolism, *n* (%)	1 (0.08)	4 (0.32)	0.177
Minor bleeding, *n* (%)	19 (1.53)	8 (0.65)	0.035
The combined secondary endpoint, *n* (%)	31 (2.50)	35 (2.83)	0.620

Deep vein thrombosis occurred more frequently in the non-bridging group (1.21% vs. 0.4%, *p* = 0.024), and there was a trend toward more pulmonary embolism in the non-bridging group (0.32% vs. 0.08%, *p* = 0.177). Minor bleeding occurred in 0.65% of the patients in the non-bridging group and in 1.53% of the patients in the bridging group (*p* = 0.035), which indicated that LMWH bridging therapy increased the risk of perioperative minor bleeding. However, there was no significant difference between the groups in the incidence of the combined secondary endpoint (*p* = 0.620).

The multivariable analysis showed that LMWH bridging therapy (RR 3.114 [95% CI 1.124–8.626]), creatinine clearance < 30 mL/min (RR 3.931 [95% CI 1.121–13.787]), perioperative mean arterial pressure < 60 mmHg (RR 1.416 [95% CI 1.041–1.927]), preoperative hemoglobin < 10 g/dL (RR 2.205 [95% CI 1.228–7.109]), and diabetes mellitus (RR 4.901 [95% CI 2.816–13.758]) were significantly associated with an increased risk of 30-day ischemic myocardial events, including MI and myocardial injury (Table 4). LMWH bridging therapy was significantly associated with an increased risk of minor bleeding (RR 6.560 [95% CI 1.748–14.616]) and a decreased risk of deep vein thrombosis (RR 0.119 [95% CI 0.031–0.453]). A preoperative platelet count < 70 × 10^9^/L (RR 1.732 [95% CI 1.036–2.909]) was an independent predictor of minor bleeding events (Table 5).

**Table 4:** Predictors of 30-day ischemic myocardial events in multivariate analyses

	Multivariate analysis		*P* value
	RR	95% CI	*P*
Age	0.952	(0.833, 1.060)	0.282
Gender	1.137	(0.351, 3.145)	0.817
ASA			
I–II	Reference		
III–IV	1.061	(0.291, 3.821)	0.928
BMI (kg/m^2^)			
18–25	Reference		
25–30	0.572	(0.175, 1.874)	0.357
> 30	1.633	(0.217, 3.826)	0.635
LMWH bridging			
No bridging	Reference		
Bridging	3.114	(1.124, 8.626)	0.029
Creatinine clearance (ml/min)			
> 60	Reference		
30–60	0.352	(0.056, 2.221)	0.267
< 30	3.931	(1.121, 13.787)	0.032
Perioperative MAP (mmHg)			
< 60	1.416	(1.041, 1.927)	0.027
60–70	Reference		
> 70	0.954	(0.821, 1.109)	0.541
Preoperative hemoglobin(g/dl)			
> 12	Reference		
10–12	0.359	(0.048, 2.703)	0.320
< 10	2.205	(1.228, 7.109)	0.019
Preoperative platelet(×10^9^/L)			
> 300	0.903	(0.835, 1.052)	0.073
100–300	Reference		
70–100	1.156	(0.415, 3.210)	0.785
< 70	1.331	(0.552, 3.589)	0. 358
Diabetes mellitus	4.901	(2.816, 13.758)	0.006

*MAP* mean arterial pressure

**Table 5:** Predictors of 30-day minor bleeding outcomes in multivariate analyses

	Multivariate analysis		*P* value
	RR	95% CI	*P*
Age	0.416	(0.089, 1.949)	0.266
Gender	0.572	(0.175, 1.874)	0.357
ASA			
I–II	Reference		
III–IV	0.413	(0.043, 4.004)	0.446
BMI (kg/m^2^)			
18–25	Reference		
25–30	0.631	(0.265, 1.729)	0.471
> 30	1.528	(0.183, 2.395)	0.552
LMWH bridging			
No bridging	Reference		
Bridging	6.560	(1.748–14.616)	0.005
Creatinine clearance (ml/min)			
> 60	Reference		
30–60	0.397	(0.085, 1.764)	0.236
< 30	0.468	(0.059, 2.708)	0.322
Perioperative MAP (mmHg)			
< 60	1.020	(0.523, 2.386)	0.964
60–70	Reference		
> 70	0.930	(0.755, 1.295)	0.781
Preoperative platelet(×10^9^/L)			
> 300	0.981	(0.817, 1.179)	0.834
100–300	Reference		
70–100	1.060	(0.892, 1.261)	0.506
< 70	1.732	(1.036, 2.909)	0.038
Diabetes mellitus	2.874	(0.788, 5.968)	0.432

## Discussion

The present study shows that LMWH bridging therapy did not increase the occurrence of the composite outcome of perioperative acute MI, cardiac death, or stroke at 30 days in patients who underwent surgery > 1 year after PCI compared with placebo. However, compared with placebo, bridging therapy decreased the incidence of perioperative myocardial injury after surgery. Compared with the bridging group, the non-bridging group had a significantly higher proportion of patients treated for two-vessel disease and a lower proportion of patients treated for single-vessel disease. This result may be one of the reasons for the higher incidence of perioperative myocardial injury in the non-bridging group. Moreover, LMWH bridging therapy did not increase the risk of perioperative major bleeding, and it decreased the occurrence of perioperative deep vein thrombosis. Perioperative antithrombotic management is based on risk assessment of thromboembolism and bleeding. Previous studies have been published regarding LMWH bridging in patients with implanted coronary stents undergoing surgery, but the results contradict our findings [4, 13, 25, 26]. This inconsistency may be explained as follows. First, the mean time interval between PCI and surgery was 75 months in the present study. However, 30.3% of the included patients underwent surgery within 180 days after PCI. Surgery within 1 year of PCI is associated with an increased risk of perioperative major adverse cardiovascular events (MACEs) [27–30]. After 1 year of PCI, the rates of death, MI, and stent thrombosis return to baseline [29, 31, 32]. Second, age has long been established as a factor influencing drug pharmacokinetics. Elderly patients have a higher incidence of age-related comorbidities, less lean body mass, and an increased bleeding risk [33–35]. Thus, the LMWH dose in the present study was reduced (dalteparin sodium 2500 IU subcutaneously twice daily). Low-dose LMWH is likely to achieve much of the benefit of therapeutic-dose anticoagulation while minimizing the risk of postoperative major bleeding [6, 36]. Third, the present study showed that LMWH can be safely resumed 24 h after surgery/procedures with a low-to-moderate bleeding risk and 48–72 h after surgery/procedures with a high bleeding risk. The appropriate time window for resuming LMWH is very important. If there is no contraindication after fully evaluating the risk of bleeding and thrombosis, perioperative use of low-dose LMWH will help reduce the risk of perioperative lower-limb venous thrombosis.

The presence of coronary artery disease is associated with an increased risk of postoperative cardiovascular complications [37, 38]. In particular, surgical procedures are performed in patients with a history of PCI, in whom the risk of perioperative adverse cardiac events is potentially is potentially high [39, 40]. Many older patients are treated with antiplatelet agents for the secondary prevention of cardiovascular events. However, the administration of antiplatelet agents (most commonly aspirin) increases the risk of major perioperative bleeding. The balance between bleeding and thrombotic risk related to perioperative maintenance of antiplatelet therapy should be outweighed [7, 41, 42]. To date, studies guiding perioperative antiplatelet management in patients with coronary stents have shown inconsistent results. Clinicians often have difficulty choosing a management strategy that allows the surgery to be as safe as possible while minimizing the risk of perioperative cardiac events. Previous studies have reported that antiplatelet therapy confers an increased risk of bleeding [43–46] and predicts poor outcomes [47]. Real-world clinical practice and published studies have reported perioperative discontinuation of antiplatelet therapy after PCI [3, 48, 49]. At present, no specific recommendations have been made for the use of LMWH for bridging patients on antiplatelet therapy to surgery. In real life, perioperative discontinuation of antiplatelet therapy and bridging with LMWH are common practices. In the present study, perioperative bridging therapy of patients with coronary stents implanted > 12 months before non-cardiac surgery decreased the incidence of myocardial injury and perioperative deep vein thrombosis compared with placebo. Moreover, it did not increase the risk of perioperative acute MI, cardiac death, stroke, or major bleeding. Therefore, an alternative approach might be the use of perioperative bridging therapy with LMWH, which was studied in patients who underwent PCI > 12 months in the present trial.

These ischemic myocardial events were associated with five preoperative risk factors: LMWH bridging, creatinine clearance < 30 mL/min, preoperative hemoglobin < 10 g/dL, diabetes mellitus, and perioperative mean arterial pressure. Bleeding events were associated with four preoperative risk factors: LMWH bridging, creatinine clearance < 30 mL/min, and preoperative platelet count < 70 × 10^9^/L. As shown in previous studies, perioperative adverse events were associated with high-risk patients, such as those with diabetes mellitus [50, 51], preoperative renal insufficiency [52], anemia [53], antiplatelet/anticoagulant therapy [54, 55], and a low platelet count [56]. Clinicians should consider risk factors when deciding whether to discontinue perioperative antiplatelet therapy or use LMWH bridging therapy in the perioperative period.

Defining the trade-off between ischemia and bleeding requires not only an understanding of the thrombotic risk of the patient (usually defined by cardiologists) but also a clear understanding of the unique bleeding risk of each surgical procedure, which requires the professional knowledge of the surgeon. According to existing guidelines, perioperative management of antithrombotic therapy should be discussed between the surgeon and the cardiologist [13, 57, 58].

Several studies have shown that the risk of perioperative MACEs is dependent on the time from PCI to non-cardiac surgery [29, 59, 60]. However, the optimal timing for non-cardiac surgery after PCI still remains controversial. There are data suggesting that DESs may have a greater risk of late stent thrombosis than BMSs beyond 12 months after implantation, particularly in the perioperative period [61].

This study had several limitations. First, this study was a single-center study and most of the included patients were male, which could have led to selection bias. Second, the results should not be applied to patients undergoing cardiac surgery, who were specifically not enrolled in this clinical trial. Third, LMWH has different mechanisms of action to antiplatelet therapy; therefore, it is not officially recommended for bridging because it cannot substitute the effects of aspirin or P2Y12 inhibitors. However, in patients with coronary stents undergoing non-cardiac surgery, LMWH bridging therapy is frequently used in clinical practice until the previous antiplatelet regimen can be resumed. Against this background, the present trial was designed to compare the clinical benefits and risks of discontinuing antiplatelet drugs with LMWH bridging therapy. Fourth, numerous other factors, such as functional status, comorbidity, mental health status [62], and duration of surgery [63], may have confounded the relationship between age and clinical outcomes [64]. Additionally, frailty may affect a patient’s ability to tolerate, survive, and eventually recover from surgical stress [65]. Therefore, the effect of age alone may be difficult to determine unless these other factors are considered. Fifth, the independent predictors of the clinical adverse endpoints are consistent with the results reported in previous literature [50–56]. Therefore, this study did not evaluate predictive factors or establish a validation queue for cross validation of risk factors to prove the predictive capability.

## Conclusions

This randomized placebo-controlled trial provides an update on the practical recommendations for perioperative antithrombotic management in patients treated with coronary stents > 12 months in non-cardiac surgery according to the predicted individual risk of thrombotic complications against the anticipated risk of surgical bleeding. The study demonstrated the safety and efficacy of perioperative LMWH bridging therapy in elderly patients with coronary stents implanted > 1 year before undergoing non-cardiac surgery. Perioperative discontinuation of antiplatelet therapy and bridging with half-dose LMWH is relatively safe and effective.

### Supplementary Information

**Supplementary Material 1.**

## Data Availability

All of data and materials were presented within the manuscript.

## Abbreviations

APTT: Activated partial thromboplastin time
ASA: American society of anesthesiologists
BARC: Bleeding academic research consortium
BMI: Body mass index
BMS: Bare metal stent
CIs: Confidence intervals
CK: Creatine kinase
CONSORT: Consolidated standards of reporting trials
DES: Drug-eluting stents
ECG: Electrocardiogram
hs-cTnT: High-sensitivity cardiac troponin T
ISTH: International society on thrombosis and haemostasis
LMWH: Low-molecular-weight heparin
MACE: Major adverse cardiovascular events
MAP: Mean arterial pressure
MI: Myocardial infarction
NT-proBNP: N-terminal pro-brain natriuretic peptide
PCI: Percutaneous coronary intervention
RCRI: Revised cardiac risk index
RR: Relative risk
ST: Stent thrombosis
