# Supplementary figures and images for: Impact of perioperative low-molecular-weight heparin therapy on clinical events of elderly patients with prior coronary stents implanted > 12 months undergoing non-cardiac surgery: a randomized, placebo-controlled trial
Source: BMC Med. 2024 Apr 23;22:171. doi: 10.1186/s12916-024-03391-2 (PMC11036782; doi:10.1186/s12916-024-03391-2)

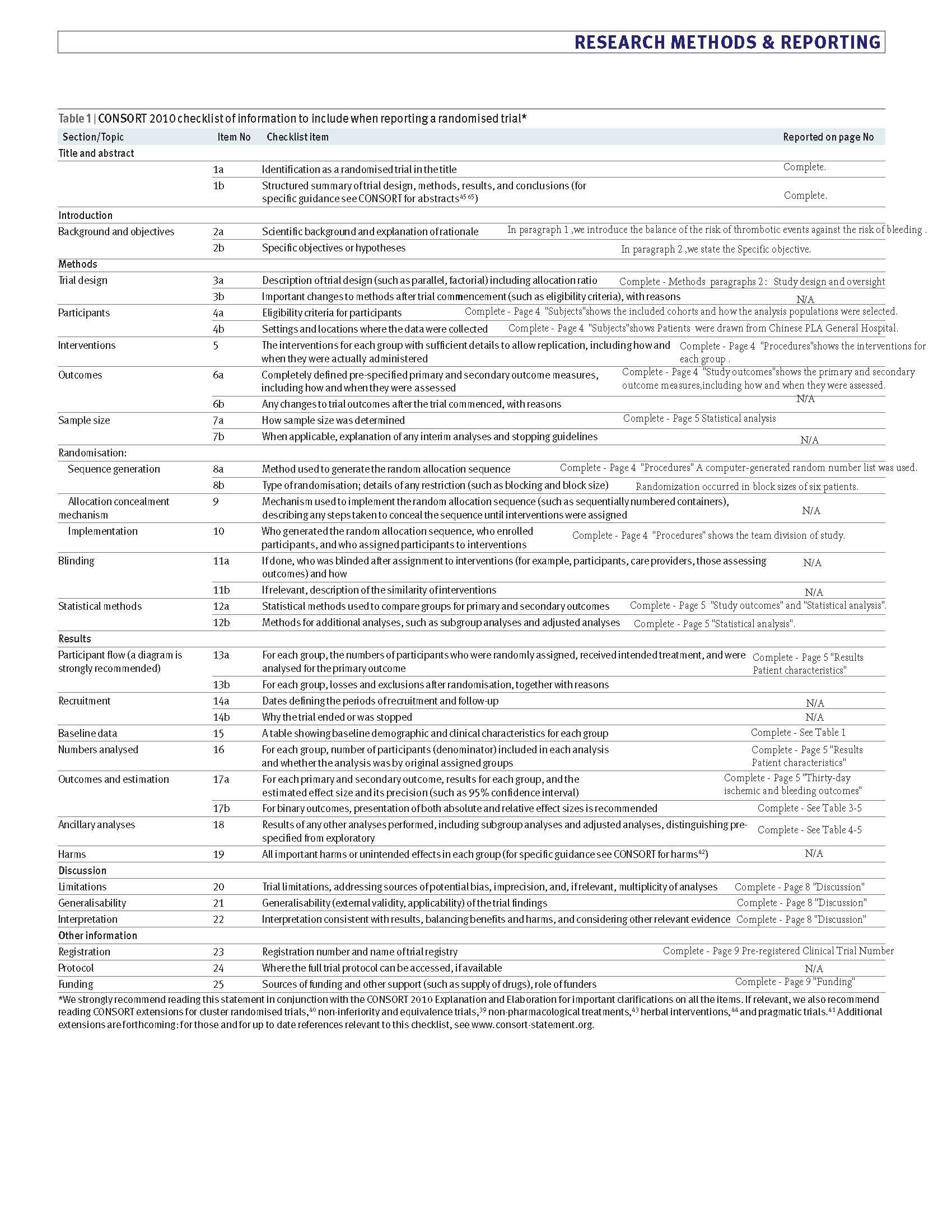

Supplement: Supplementary file 1 — Supplementary Material 1. [file 12916_2024_3391_MOESM1_ESM.jpg]
